# Task shifting roles, interventions and outcomes for kidney and cardiovascular health service delivery among African populations: a scoping review

**DOI:** 10.1186/s12913-023-09416-5

**Published:** 2023-05-05

**Authors:** Ikechi G. Okpechi, Ijezie I. Chukwuonye, Udeme Ekrikpo, Jean Jacques Noubiap, Yemi R. Raji, Yusuf Adeshina, Samuel Ajayi, Zunaid Barday, Malini Chetty, Bianca Davidson, Emmanuel Effa, Stephen Fagbemi, Cindy George, Andre P. Kengne, Erika S. W. Jones, Hamidu Liman, Mohammad Makusidi, Hadiza Muhammad, Ikechukwu Mbah, Kwazi Ndlovu, Grace Ngaruiya, Chimezie Okwuonu, Ugochi Samuel-Okpechi, Elliot K. Tannor, Ifeoma Ulasi, Zulkifilu Umar, Nicola Wearne, Aminu K. Bello

**Affiliations:** 1 Department of Medicine, University of Alberta, Edmonton, Canada; 2 Division of Nephrology and Hypertension, University of Cape Town, Cape Town, South Africa; 3 Department of Internal Medicine, Federal Medical Centre, Umuahia, Abia State Nigeria; 4 Division of Nephrology, University of Uyo, Akwa Ibo State, Uyo, Nigeria; 5 Division of Cardiology, Department of Medicine, University of California-San Francisco, San Francisco, CA USA; 6 Department of Medicine, University of Ibadan, Ibadan, Oyo State Nigeria; 7 Division of Nephrology, Usmanu Danfodiyo University Teaching Hospital, Sokoto, Nigeria; 8 Department of Medicine, University of Calabar, Calabar, Nigeria; 9 Department of Internal Medicine, Edward Francis Small Teaching Hospital, Banjul, The Gambia; 10 Department of Epidemiology, Ondo State Ministry of Health, Ondo, Nigeria; 11 Non-Communicable Disease Research Unit, South Africa Medical Research Council, Cape Town, South Africa; 12 Dept of Medicine College of Med and Health Sciences, Bingham University, Jos, Nigeria; 13 School of Nursing, Mount Kenya University, Thika, Kenya; 14 Department of Internal Medicine, Federal Medical Centre, Abuja, Nigeria; 15 Department of Medicine, Kwame Nkrumah University of Science and Technology, Kumasi, Ghana; 16 Department of Medicine, University of Nigeria, Ituku Ozalla, Enugu State Nigeria

**Keywords:** Africa, Cardiovascular disease, Chronic kidney disease, Diabetes, Hypertension, Health workforce, Task shifting

## Abstract

**Background:**

Human resources for health (HRH) shortages are a major limitation to equitable access to healthcare. African countries have the most severe shortage of HRH in the world despite rising communicable and non-communicable disease (NCD) burden. Task shifting provides an opportunity to fill the gaps in HRH shortage in Africa. The aim of this scoping review is to evaluate task shifting roles, interventions and outcomes for addressing kidney and cardiovascular (CV) health problems in African populations.

**Methods:**

We conducted this scoping review to answer the question: “what are the roles, interventions and outcomes of task shifting strategies for CV and kidney health in Africa?” Eligible studies were selected after searching MEDLINE (Ovid), Embase (Ovid), CINAHL, ISI Web of Science, and Africa journal online (AJOL). We analyzed the data descriptively.

**Results:**

Thirty-three studies, conducted in 10 African countries (South Africa, Nigeria, Ghana, Kenya, Cameroon, Democratic Republic of Congo, Ethiopia, Malawi, Rwanda, and Uganda) were eligible for inclusion. There were few randomized controlled trials (*n* = 6; 18.2%), and tasks were mostly shifted for hypertension (*n* = 27; 81.8%) than for diabetes (*n* = 16; 48.5%). More tasks were shifted to nurses (*n* = 19; 57.6%) than pharmacists (*n* = 6; 18.2%) or community health workers (*n* = 5; 15.2%). Across all studies, the most common role played by HRH in task shifting was for treatment and adherence (*n* = 28; 84.9%) followed by screening and detection (*n* = 24; 72.7%), education and counselling (*n* = 24; 72.7%), and triage (*n* = 13; 39.4%). Improved blood pressure levels were reported in 78.6%, 66.7%, and 80.0% for hypertension-related task shifting roles to nurses, pharmacists, and CHWs, respectively. Improved glycaemic indices were reported as 66.7%, 50.0%, and 66.7% for diabetes-related task shifting roles to nurses, pharmacists, and CHWs, respectively.

**Conclusion:**

Despite the numerus HRH challenges that are present in Africa for CV and kidney health, this study suggests that task shifting initiatives can improve process of care measures (access and efficiency) as well as identification, awareness and treatment of CV and kidney disease in the region. The impact of task shifting on long-term outcomes of kidney and CV diseases and the sustainability of NCD programs based on task shifting remains to be determined.

**Supplementary Information:**

The online version contains supplementary material available at 10.1186/s12913-023-09416-5.

## Introduction

The low availability of human resources for health (HRH) is a major limitation to equitable access to healthcare in Africa [1–4]. African countries have the most severe shortage of HRH in the world with > 60% of countries experiencing extreme shortage of HRH located in the region [5]. It is estimated that although Africa bears 24% of the global disease burden, it has only 3% of the world’s health workforce and < 1% of the world’s financial resources for health [6]. Data from the World Health Organization (WHO) projects that Africa will have the lowest total stock of HRH (physicians, nurses, midwives, and other cadres of health workers) by 2030 and the highest increase in shortages (45% from 2013) than other world regions [7]. A global survey on the availability of HRH for kidney care showed massive disparities between world regions [4]. With a median of 0.62 (interquartile range [IQR]: 0.24–1.56) nephrologists per million population [pmp], Africa had the lowest distribution of nephrologists compared to other regions such as Western Europe (24.36 [IQR: 18.07–29.91] nephrologists pmp) [4]. Africa also had one of the lowest numbers of nephrology trainees and reported higher shortages of HRH for other cadres of kidney care providers (e.g., dialysis nurses, dialysis technologists, kidney transplant coordinators, access surgeons, etc.) than other regions [4].

The causes of HRH shortage in sub-Saharan Africa include existing shortfalls in pre-service training, international migration (brain drain), career changes among health workers, premature retirement, morbidity, and premature mortality [8–10]. Task shifting[11] which involves the rational redistribution of tasks from highly qualified health workers to health workers with shorter training or fewer qualifications could be useful for improving healthcare services for non-communicable diseases (NCDs) including kidney and cardiovascular (CV) health services. Task shifting has been used extensively in communicable diseases health service delivery and shown to be effective, acceptable and associated with increased access to treatment, cost-effectiveness, improved quality of care, and improved health outcomes [12–15]. Studies on task-shifting for NCDs care delivery have also shown efficacy in improving outcomes. Interventions with nurse-led diabetes and hypertension care led to significant reductions in pooled glycated hemoglobin (HbA1c) of − 0·54% (95% CI − 0·89 to − 0·18; *P* < 0.0001)[16] as well as significant reductions in pooled systolic blood pressure (BP) of –5∙34 mm Hg (95% CI –9∙00 to –1∙67; *P* < 0.01) [17]. However, they only included randomized controlled trials (RCTs) and therefore very few African studies due to lack of such RCTs in Africa [18]. Despite these benefits, task shifting has been associated with several negative impacts including staff conflicts [19], malpractice, and quackery[20] among others.

International stakeholder organizations (e.g., WHO) have put forward recommendations on task shifting as a measure towards addressing workforce shortages in low-income and lower-middle-income countries (LMICs). We therefore aimed to conduct a scoping literature review to evaluate task shifting roles, interventions, and outcomes for addressing kidney and CV health problems in African populations.

## Methods

We developed and conducted this review using the methodology of the Preferred Reporting Items for Systematic Reviews and Meta-Analyses extension for Scoping Reviews (PRISMA-ScR) [21, 22]. We also leveraged the six-stage methodological framework developed by Arksey and O’Malley[23] in formulating the study protocol.

### Information sources and search strategy

The search strategy was developed to ensure that a comprehensive review of the existing evidence base was achieved, and we searched Medline (Ovid), Embase (Ovid), CINAHL, ISI Web of Science, and Africa journal online (AJOL). Additional hand searches were carried out by citations tracking and reference chaining of identified studies. The search strategies are as shown in Supplementary Table S1.

### Eligibility criteria

We included studies that met the following characteristics:

• Studies performed in adult Africans (aged ≥ 18 years) and focused on kidney or CV risk reduction.
• Studies in which the intervention used task shifting/sharing to non-physician healthcare workers [e.g., nurses, pharmacists, community health workers, etc.] and involved screening/detection of kidney disease or CV disease, patient education/counselling, prescribing of medications, or methods to improve treatment adherence.
• Studies reporting outcomes of task shifting/sharing interventions related to kidney disease (e.g., change in proteinuria, or glomerular filtration rate) or improved CV risk factors (e.g., improved BP, glycaemic index, serum lipids, weight, etc.).
• Studies reporting improved quality initiatives for CV and kidney care (medication adherence, awareness, or clinic attendance).
• Experimental, quasi-experimental, or observational studies.
• Published in English.
• Study period: from inception to 30^th^ June 2021.

The following study types were excluded:

• Task shifting interventions for communicable diseases (e.g., HIV, Malaria, etc.) or for conditions not related to kidney or CV risks (e.g., maternal and child health, mental health, etc.).
• Studies focused on implementation, training, barriers or facilitators of using various workforce for task-shifting for care.
• Studies on Africans not conducted within the African continent.
• Review articles, editorials, commentaries, letters to the editor, and guidelines or recommendations on task shifting.

Although kidney disease was reported as defined in each study, we defined it in this study as the assessment of participants with acute kidney injury (AKI), chronic kidney disease (CKD), kidney failure, or asymptomatic urinary abnormalities (hematuria and/or proteinuria).

**Fig. 1:**
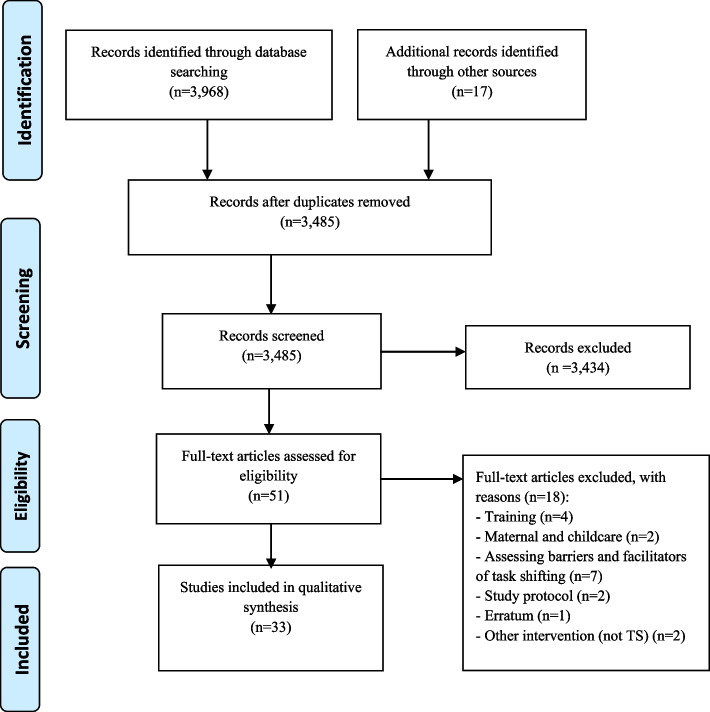
PRISMA Flow Diagram for study selection

Two reviewers (IIC and YRR) independently screened all identified citations for potential inclusion and a third reviewer (IGO) was consulted for resolution when agreement on a citation could not be reached. The review process first involved screening of the titles and abstracts and then a detailed review of all selected full texts to ascertain eligibility for inclusion (Fig. 1).

### Data items and data abstraction process

All relevant information from selected studies was summarized and collated in a Microsoft Excel spreadsheet. We collected data on the study characteristics (i.e., year of publication, sample size of the study, country of the study, study design, and study setting), intervention utilized i.e., type of HRH task was shifted to (e.g., nurse, pharmacist, community health worker, others), role of HRH (triage/referral, screening/detection, education/counselling, management), disease type for task shifting (hypertension, diabetes, hyperlipidemia, obesity, and kidney disease), and outcomes reported including improved BP, glycaemic markers, kidney function, serum lipids, weight reduction, etc. The impact of task shifting was assessed as any report of improved kidney function or CV risk factors (i.e., reduced BP, glycaemic levels, serum lipids, body mass index (BMI), proteinuria or increase in estimated glomerular filtration rate), and any reported increase in quality initiatives (i.e., increase in medication adherence, awareness, or clinic attendance).

### Collating, summarizing, and reporting of the results

All extracted data were reviewed for accuracy and completeness. We followed recommendations to extend the scoping review process by adding thematic analysis[24] and the data were analyzed qualitatively using both deductive (pre-identified themes) and inductive (new identified themes) approaches. Most data were captured as “yes” or “no” with the proportions of “yes” responses descriptively reported as counts and percentages. The number of studies assigned to each worker category for a specific task was used as denominator in assessing outcomes within such task. For example, the number of studies for which tasks were shifted to nurses for hypertension was used to assess the proportion with improved BP levels for nurses. This was also done for other worker categories. Using 2013 WHO data on workforce densities for universal health coverage (UHC) and sustainable development goals [25], we also estimated the ratio of nurses to physicians as well as all other cadres of health workforce to physicians for each WHO region.

### Risk of bias assessment or quality appraisal

Following guidance on scoping review conduct, we did not perform a risk of bias assessment or quality appraisal for included studies using standard criteria [21, 22].

### Consultation exercise

Consultation was not conducted as part of this study.

### Patient and public involvement

Patients and the public were not involved in this scoping review.

## Results

### Overall features of included studies

**Fig. 2:**
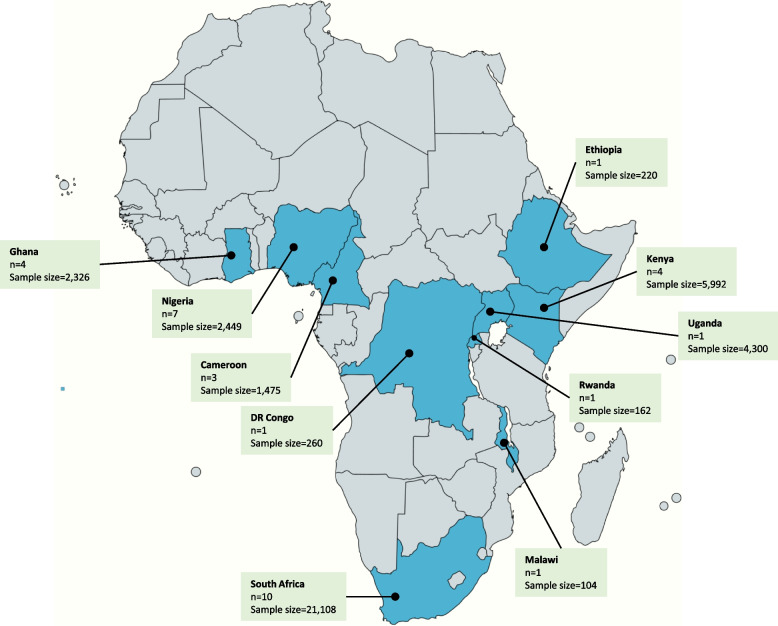
Map of Africa showing included countries, number of studies and sample size per country. (Created using: www.mapchart.net)

Our initial search identified 3,968 studies of which 33 studies conducted in 10 African countries: South Africa (*n* = 10; 30.3%) [26–35], Nigeria (*n* = 7; 21.2%) [36–42], Ghana (*n* = 4; 12.1%) [43–46], Kenya (*n* = 4; 12.1%) [47–50], Cameroon (*n* = 3; 9.1%) [51–53], Democratic Republic of Congo (*n* = 1; 3.0%) [54], Ethiopia (*n* = 1; 3.0%) [55], Malawi (*n* = 1; 3.0%) [56], Rwanda (*n* = 1; 3.0%) [57], and Uganda (*n* = 1; 3.0%) [58], were deemed eligible for inclusion (Table 1 and Fig. 2). More studies were conducted in both rural and urban areas (*n* = 13; 39.4%) than in urban areas only (*n* = 12; 36.4%) or rural areas only (*n* = 8; 24.2%). RCTs were the least employed study design included (*n* = 6; 18.2%)[29, 31, 33, 37, 42, 43] with half of these conducted in South Africa (*n* = 3; 50.0%). The disease of focus for included studies was hypertension only (*n* = 16; 48.5%) [26, 33, 36–39, 41, 43, 45, 46, 49, 50, 52, 54, 57, 58], diabetes mellitus only (*n* = 5; 15.2%) [27–30, 55], acute kidney injury (*n* = 1; 3.0%) [56], and multiple NCD risk factors (*n* = 11; 33.3%) [31, 32, 34, 35, 40, 42, 44, 47, 48, 51, 53].

**Table 1:** Demographic features of included studies

First author [Ref}	Publication year	Country of study	Study design	Study setting	Sample size	Female (%)	Study duration (months)	Population/disease condition	Task shifted to:
Coleman et al. [24]	1998	South Africa	Observational prospective	Rural	1343	66.7	24	Hypertension	Nurses
Gill et al. [25]	2008	South Africa	Observational prospective	Urban	284	NR	18	Diabetes	Nurses
Price et al. [26]	2011	South Africa	Observational prospective	Rural	80	70.0	48	Diabetes	Nurses
Mash et al. [27]	2014	South Africa	RCT	Rural	1570	73.8	12	Diabetes	Health promoters
Muchiri et al. [28]	2015	South Africa	Observational prospective	Rural	62	86.6	12	Diabetes	Nurses
Fairall et al. [29]	2016	South Africa	RCT	Urban + Rural	4,393	73.0	14	Hypertension, Diabetes	Nurses
Morris-Paxton et al. [30]	2018	South Africa	Observational prospective	Rural	1885	NR	38	Hypertension, Diabetes, CVD	CHW
Rampamba et al. [31]	2019	South Africa	RCT	Urban + Rural	86	86.1	6	Hypertension	Pharmacists
Madela S et al. [32]	2020	South Africa	Observational prospective	Urban + Rural	10 832	71.9	12	Hypertension, Diabetes, CVD	CHW
Sharp et al. [33]	2022	South Africa	Observational prospective	Urban + Rural	573	78.0	18	Hypertension, Diabetes, CVD	Nurses
Oparah et al. [34]	2006	Nigeria	Observational prospective	Urban	36	27.0	6	Hypertension	Pharmacists
Adeyemo et al. [35]	2013	Nigeria	RCT	Urban + Rural	544	NR	6	Hypertension	Nurses
Nelissen et al. [36]	2018	Nigeria	Cross-sectional	Urban	236	58.8	13	Hypertension	Pharmacists
Ozoememna et al. [37]	2019	Nigeria	Observational prospective	Urban	400	50.3	4	Hypertension	Nurses
Amadi et al. [38]	2020	Nigeria	Cross-sectional	Urban	889	57.4	3	Hypertension, Diabetes, CVD	Pharmacists
Ojji et al. [39]	2020	Nigeria	Observational prospective	Urban	60	65.0	1	Hypertension	CHW
Onyinye et al. [40]	2021	Nigeria	RCT	Urban	284	49.7	9	Hypertension, Diabetes	Pharmacists
Sarfo et al. [41]	2015	Ghana	RCT	Urban	60	70.0	9	Hypertension	Nurses
Marfo et al. [42]	2016	Ghana	Observational prospective	Urban + Rural	170	59.0	13	Hypertension, Diabetes, CVD	Pharmacists / MCA
Ogedegbe et al. [43]	2018	Ghana	Observational prospective	Urban + Rural	757	60.2	47	Hypertension	Nurses
Adler et al. [44]	2019	Ghana	Observational prospective	Urban	1339	69.2	12	Hypertension	Nurses
Some et al. [45]	2016	Kenya	Cross-sectional	Urban	616	72.0	4	Hypertension, Diabetes, Others*	Nurses
Mannik et al. [46]	2018	Kenya	Cross-sectional	Rural	2865	55.0	22	Hypertension, Diabetes, CVD	CHW
Vendanthan et al. [47]	2019	Kenya	Cross-sectional	Urban + Rural	1460	58.0	15	Hypertension	CHW
Vendanthan et al. [48]	2020	Kenya	Cross-sectional	Urban + Rural	1051	65.0	12	Hypertension	Nurses
Kengne et al. [49]	2009	Cameroon	Observational prospective	Urban + Rural	225	42.7	NR	Hypertension, Diabetes, CVD	Nurses
Kengne et al. [50]	2009	Cameroon	Observational prospective	Urban + Rural	454	47.4	26	Hypertension	Nurses
Labhardt et al. [51]	2010	Cameroon	Observational prospective	Rural	796	69.0	24	Hypertension, Diabetes	Non-physician clinician
Lulebo et al. [52]	2017	DRC	Cross-sectional	Urban	260	53.1	24	Hypertension	Nurses
Hailu et al. [53]	2018	Ethiopia	Observational prospective	Urban + Rural	220	35.2	9	Diabetes	Nurses
Kirwan et al. [54]	2016	Malawi	Observational prospective	Urban	104	51.9	3	AKI	Nurses
Ngoga et al. [55]	2019	Rwanda	Observational prospective	Urban + Rural	162	79.6	12	Hypertension	Nurses
Stephens et al. [56]	2021	Uganda	Cross-sectional	Rural	4,300	78.0	120	Hypertension	Others

Abbreviations: *DRC* Democratic republic of congo, *CVD* Cardiovascular disease risk factors, *NR* Not reported, *NA* Not applicable, *RCT* Randomized control trial, *AKI* Acute kidney injury, *CHW* Community health workers, *MCA* Medicine counter attendants

^*^ SCD, Asthma, Epilepsy

Overall, tasks were mostly shifted to nurses (*n* = 19; 57.6%) [26–28, 30, 31, 35, 37, 39, 43, 45–47, 50–52, 54–57], and this was true for studies from South Africa, Ghana, Cameroon, Democratic Republic of Congo (DRC), Ethiopia, Malawi and Rwanda. However, in four out of the seven studies from Nigeria, more tasks were shifted to pharmacists[36, 38, 40, 42] (Supplementary Table S2). Several studies reported multiple roles for tasks to be shifted and the roles included treatment and adherence support (*n* = 28; 84.9%) [26–37, 39, 41, 42, 44–47, 49, 51–57], disease screening and detection (*n* = 24; 72.7%) [26, 27, 30–32, 34, 35, 37, 38, 40, 41, 44–46, 48–53, 55–58], education and counselling (*n* = 24; 72.7%) [26–30, 32–34, 36–39, 41–46, 51–53, 55–57], and triage (*n* = 13; 39.4%) [26, 30, 32–34, 40, 41, 44, 47–49, 58]. In each category of task shifted roles examined, studies from South Africa had the highest proportions (Supplementary Table S2). Summaries of study objectives, interventions, results, and outcomes are provided in Supplementary Table S3.

### Task shifting for hypertension

**Fig. 3:**
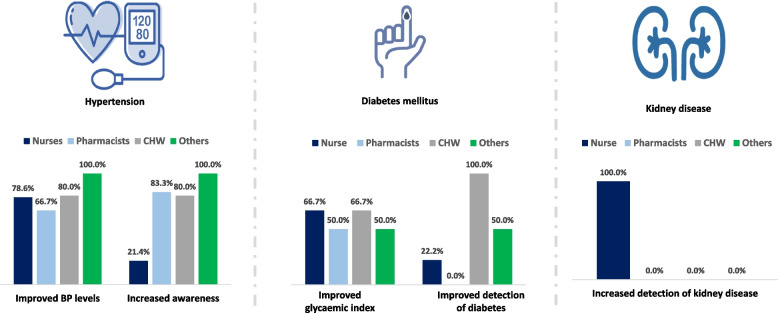
Relationship between task shifting and clinical outcomes. Abbreviations: CHW – community health workers *Others include health promoters, medicine counter assistant, non-physician clinician

Task shifting was used for hypertension (alone or with other NCD risk factor) in 27 studies (81.8%) [26, 31–54, 57, 58], and were mostly shifted to nurses (*n* = 14; 51.9%). Overall, improved BP levels and improved awareness were reported in 21 (77.8%)[30–36, 38, 41, 43–46, 49, 51–55, 57, 58] and 14 (51.9%)[32–34, 36, 38, 40, 41, 44–46, 48, 52, 53, 58] studies, respectively (Table 2). In studies where BP-related tasks were shifted, improved BP levels was reported in 78.6% (nurses; *n* = 11) [30, 31, 35, 43, 45, 46, 51, 52, 54, 55, 57], 66.9% (pharmacists; *n* = 4) [33, 36, 38, 44], 80.0% (CHW; *n* = 4) [32, 34, 41, 49], and 100% (other workers [health promoters, medicines counter assistant, or non-physician clinician]; *n* = 2)[53, 58] (Table 2 and Fig. 3). However, the proportion of studies that reported improved hypertension awareness by type of health worker was 83.3% (pharmacists; *n* = 5) [33, 36, 38, 40, 44], 80.0% (CHW; *n* = 4) [32, 34, 41, 48], 21.4% (nurses; *n* = 3) [45, 46, 52], and 100% (others; *n* = 2) [53, 58].

**Table 2:** Impact of task shifting on outcomes based on healthcare worker

**Variables (yes)**	**All *n* = 33**	**Nurses [n (%)]**	**Pharmacists [n (%)]**	**CHW [n (%)]**	**Others‡ [n (%)]**
**Task shifting role**					
- Triage	13 (39.4)	4 (30.8)	3 (23.1)	5 (38.5)	1 (7.7)
- Education/counselling	24 (72.7)	14 (58.3)	5 (20.8)	3 (12.5)	3 (9.1)
- Screening	24 (72.7)	14 (58.3)	3 (12.5)	5 (20.8)	2 (8.3)
- Management / adherence	28 (84.9)	17 (60.7)	5 (17.9)	4 (14.3)	2 (7.1)
**Hypertension [n (%)]**					
- Task shifting for hypertension	27 (81.8)	14 (51.9)	6 (23.1)	5 (19.2)	2 (7.7)
- Proportion with improved BP levels	21 (77.8)	11 (78.6)	4 (66.7)	4 (80.0)	2 (100.0)
- Proportion with improved awareness	14 (51.9)	3 (21.4)	5 (83.3)	4 (80.0)	2 (100.0)
**Diabetes [n (%)]**					
- Task shifting for diabetes	16 (48.5)	9 (56.3)	2 (12.5)	3 (18.8)	2 (12.5)
- Proportion with improved glycaemic index	10 (62.5)	6 (66.7)	1 (50.0)	2 (66.7)	1 (50.0)
- Proportion with improved detection of diabetes	6 (37.5)	2 (22.2)	0 (0.0)	3 (100.0)	1 (50.0)
**Multiple risk factors #**					
**Task shifting for multiple risk factors**	11 (33.3)	4 (36.4)	3 (27.3)	3 (27.3)	1 (9.0)
**Kidney disease [n (%)]**					
- Task shifting for kidney disease	1 (3.0)	1 (100)	0 (0.0)	0 (0.0)	0 (0.0)
- Improved detection of kidney disease	1 (3.0)	1 (100)	0 (0.0)	0 (0.0)	0 (0.0)

^‡^—health promoters, medicine counter assistant, non-physician clinician

^#^—hypertension, diabetes, smoking

CHW – community health workers

### Task shifting for diabetes mellitus

Overall, and of the 16 studies that used task shifting for diabetes mellitus [27–32, 34, 35, 40, 42, 44, 47, 48, 51, 53, 55], ten (62.5%) studies reported improved glycaemic levels (either blood glucose or glycated hemoglobin)[27, 28, 30, 32, 34, 35, 40, 51, 53, 55] while six (37.5%) studies reported improved detection of diabetes[30, 32, 34, 48, 53, 55] (Table 2). Improved glycaemic levels were reported in 66.7% (*n* = 6) of nurse-led studies [27, 28, 30, 35, 51, 55], 50.0% (*n* = 1) of pharmacist-led studies [40], 66.7% (*n* = 2) of CHW-led studies [32, 34], and 50% (*n* = 1) of studies led by other health workers[53] (Fig. 3). However, detection of diabetes was reported in 22.2% (*n* = 2; nurses), 0% (pharmacists), 100% (*n* = 3; CHW), and 50% (*n* = 1; others) (Table 2).

### Task shifting for kidney diseases

Only one study, conducted in Malawi, employed task shifting principles to improve kidney outcomes [56]. In this study, nurse-led education programs resulted in improved acute kidney injury (AKI) detection, fluid charting, and recording of urine outputs (Fig. 3).

## Discussion

The findings of our study underscore the value of task shifting for NCD detection, management, and control in Africa given the rising incidence of NCD in the continent. Importantly, we identified task shifting to be linked with improved detection, awareness, and management of hypertension, diabetes mellitus, and kidney disease in Africa. Our study also showed that non-physician healthcare workers in Africa can engage in diverse roles when tasks are shifted to them suggesting that task shifted roles can be used to fill gaps from HRH shortages for NCD care in Africa.

According to the WHO [59], NCDs are responsible for 41 million deaths annually, equivalent to 71% of all deaths globally with CV diseases accounting for 17.9 million of these deaths. Detection, screening, and treatment of NCDs, as well as palliative care, are key components of the response to NCDs. The burden of disease in Africa is largely dominated by communicable diseases and those that largely affect maternal and child health [60, 61]. However, as countries continue to undergo demographic transitions, increasing NCD prevalence has been documented in Africa [59–61]. In a study that used Global Burden of Disease (GBD) data to assess the trends in NCD prevalence in sub-Saharan Africa (SSA), disability adjusted life years (DALYs) due to NCDs increased by 67·0% between 1990 (90·6 million [95% UI 81·0–101·9]) and 2017 (151·3 million [133·4–171·8]) [61]. Cardiovascular diseases were the second leading cause of NCD burden in SSA in 2017, resulting in 22·9 million (21·5–24·3) DALYs [61].

Africa lacks the physician workforce capacity to adequately address the current or projected burden of NCDs and to implement UHC. In a 2016 document of the WHO that addressed global workforce capacity, Africa had the lowest number of physicians (0.3 per 1000 population) compared to other regions or global average (1.4 per 1000 population) [25]. However, what Africa lacks in physician numbers is made up for in the number of other health workers (Table 3) [25]. Africa has the highest population weighted density ratio of nurses/midwives-to-physicians (4.0; compared to other regions [range: 1.7 to 2.3]) and also has the highest ratio for other cadres of healthcare workers-to-physicians (2.3; compared to other regions [range: 1.1 to 2.0]) [25]. This suggests an important role for non-physician healthcare workers in closing the gaps in care provision in the region due to low number of physicians on the continent.

**Table 3:** Population-weighted density of health workers (per 1000 population) by cadre

	Physicians	Nurses/Midwives	Nurse/midwives: Physician ratio	All other cadres	All other cadres: physician ratio
Global	1.4	2.9	2.1	1.8	1.3
Africa	0.3	1.2	4.0	0.7	2.3
Americas	2.1	4.8	2.3	2.7	1.3
Eastern Mediterranean	1.2	2.1	1.8	1.6	1.3
Europe	3.2	6.8	2.1	4.0	1.3
South-East Asia	0.6	1.5	2.5	1.2	2.0
Western Pacific	1.5	2.5	1.7	1.6	1.1

Data from the World Health Organization [25]

This study showed that although all cadres of non-physician health workers participated in the delivery of task shifted interventions, the effectiveness was relatively higher when provided by workers with higher levels of education (e.g., nurses and pharmacists). There was increased reporting of outcomes for tasks shifted to every category of healthcare worker assessed in this study, even though the number of studies were fewer for all categories except nurses.. Nurses are well positioned to detect, treat and refer people with NCDs as well as to provide information, education and counselling to the public on prevention of NCDs given that they are usually the point of first contact. A WHO high-level commission on NCDs has recommended that for health systems to be reoriented for chronic disease management, nurses are uniquely placed to act as effective practitioners, health coaches, spokespersons, and health educators for patients and families throughout the life course [62]. One study from India compared the performance of nurses with doctors to determine which skills are required for NCD care delivery. Despite a lower baseline, nurses had a similar attrition in knowledge after training compared to doctors implying that nurses can be trained to deliver NCD care similar to the level provided by doctors [63]. One of the included study that assessed BP management by nurse-led and clinical officer-managed patients in Kenya did not find significant differences in BP slopes after 3 months (nurse-managed patients: slope –4.95 mmHg/month; clinical officer-managed patients: slope –5.28 mmHg/month; *P* = 0.40) [50].

Our study also demonstrated the importance of other cadres of healthcare workers (pharmacists and CHWs) in NCD awareness and management as pharmacist-led programs had the highest rates of hypertension awareness (83.3%) while CHW-led programs had the highest detection rate of diabetes mellitus (100%) (Table 2 and Fig. 3). Our study findings have implications for NCD detection and management in Africa. In a regional modelling analysis[64] of contributions to preventable premature deaths in countries that have agreed to a WHO Global Action Plan for the prevention and control of NCDs 2013–2020 [65], it was reported that if current levels of prevention continued, the probability of premature mortality from four NCDs (CVD, cancers, chronic respiratory diseases and diabetes) will increase in the African region but decrease in other regions [64]. It was therefore suggested that Africa needs more aggressive interventions to combat NCDs [31]. As our study shows, task shifting initiatives could be leveraged to close the gaps in HRH shortages and improve NCD detection, awareness, and management in the region.

Furthermore, Africa has the highest gaps in the proportion of people with kidney failure needing dialysis but unable to receive it[66] and an extremely high mortality rate in adults and children with kidney failure [67]. Although cost plays a pivotal role in the care disparities and outcomes of kidney failure patients [3], very low nephrologist density has been linked to poor outcomes in the region [4, 68]. Early NCD identification with appropriate interventions (e.g., education, counselling, pharmacotherapies, etc.) delivered by non-physician healthcare workers can modify these outcomes in the region [69, 70].

There are several advantages of task shifting, including opportunity to provide accredited and standardized pre-service and in-service training to healthcare workers [11, 58], more cost-effective and quicker addition to the competencies of experienced health workers [11], increased opportunities for patients to receive care nearer home [57], enhance the primary care model of health services, reduce the burden on tertiary care, and improve referral systems. Such strategies may also come in handy in tackling public health emergencies such as pandemics, natural disasters, and re-emerging infectious diseases which remain a huge challenge in the region [71]. However, for task shifting initiatives to be successful, they should be designed and implemented with an understanding of disease burden [72], healthcare system context [3], known barriers [73], and integrated chronic disease models [74, 75]. Health system strengthening, restructuring, appropriate training, and health-care regulation are necessary prerequisites for task shifting initiatives to yield desirable results [3, 76]. For instance, task shifting for a hypertension program can be implemented using guideline concordant hypertension triage and treatment algorithms integrated with HIV care at the primary healthcare level [15]. Similarly, task shifting for kidney disease could include use of guideline concordant methods to train other cadres of health workers on referral to nephrology, for implementing quality improvement initiatives, participate in clinic tasks (e.g., urinalysis) [77], use of protocols to initiate fluid therapies in cases of community acquired AKI [78], and guideline-based checklists for management of early-stage CKD [79]. This will require in- service training, supportive supervision, and expansion of job descriptions given that barriers to task shifting may include interprofessional staff conflicts [19, 80], poor organizational leadership structures [81], professional protectionism (e.g., physicians may feel that their profession is being invaded by others) [82], malpractice, quackery [20], and issues of professional boundaries and regulations (i.e., workers may feel they won’t have legal protection for additional tasks if something went wrong). Other areas of contention include poor wages and working conditions (i.e., unwillingness to be deployed to remote areas where shortage is highest), prohibitive policies and laws (e.g., laws that prevent lower-level cadres from carrying out particular tasks), and working outside of regulated practice (with possible adverse patient outcomes) [83].

Enabling task shifting initiatives will require potent HRH policies across countries in the region. In 2012, a WHO roadmap for scaling up health workforce was endorsed by African Health Ministers and included: (i) developing health workforce policies and strategies; (ii) ensuring that all countries have increased their health workforce to a minimum density threshold of 2.3 per 1000 population; (iii) maintaining an appropriate skill mix of health workers with population-relevant competences; (iv) ensuring equitable redeployment and distribution of the health workforce; and (v) measures to attract and retain health professionals (e.g., improving remuneration, working and living conditions) [84]. However, by 2015, only 36% of countries in the African Region had a policy for HRH, although 72% had a strategic plan for HRH suggesting more efforts are still needed to ensure that such polices are implemented to reduce shortages in HRH and increase the possibilities of attaining UHC goals [85]. Implementing WHO strategies on HRH targets for 2030[7] which includes (i) optimizing performance, quality, and impact of the HRH through evidence-informed policies and strengthened health systems at all levels; (ii) aligning investment in HRH with the current and future needs of the population and health systems, (iii) building the capacity of institutions at subnational, national, regional, and global levels for effective public policy stewardship, leadership, and governance of actions on HRH; and (iv) strengthening data on HRH for monitoring and accountability of national and regional strategies will ensure that gaps in health workforce in Africa can be mitigated.

We identified some limitations to this study, including the low number of studies from most countries, low sample size of included studies and use of studies published in only the English language which could have excluded studies from francophone countries in the region. Only South Africa had sufficient numbers of studies and the largest sample size to increase the applicability of our observations; four countries had only one study included with sample sizes ranging from 104 to 4,300. Another limitation of this study is the availability of a single study to evaluate the impact of task shifting strategies on kidney outcomes. This again limits our ability to draw firm inferences on the effectiveness of such strategies for kidney care on the continent. However, given that the study showed task shifting to be useful in identifying AKI cases suggesting that such strategies could be employed for improving care and outcomes. Although none of the included studies reported any negative or unintended consequences occurring from task shifting strategies, such consequences have sometimes been reported when task shifting initiatives are utilized [83]. This can also be viewed as a limitation of this study given that our study design did not include qualitative studies which are more likely to highlight such events [20]. Even though it is possible that there were no such effects, being able to balance the risks and efficacy of task shifting for CV and kidney care could assist policy development on scope of work when task shifting strategies are employed. Notwithstanding these limitations, this study shows that task shifting strategies can be implemented in Africa for NCD detection and management.

## Conclusion

Despite the large-scale HRH challenges that are present in Africa for CV and kidney health, our data shows that task shifting initiatives can improve process of care measures such as (i) access to healthcare and efficient triage; (ii) improve risk identification; (iii) improve disease awareness; and (iv) improve treatment and management of CV and kidney disease in the region. The impact of task shifting on kidney and CV health on long term outcomes, population-level control and sustainability of programs remain to be determined. These findings have implications on the design of NCD programs for implementation of task shifting in workforce across the region or expansion of the current programs.

## Supplementary Information

**Additional file 1:** **Supplementary Table S1.** Search strategy. **Supplementary Table S2.** Summary of studies aims, interventions, results, and conclusions. **Supplementary Table S3. **Features of included studies by country.

## Data Availability

The datasets used and/or analyzed during the current study are available from the corresponding author on reasonable request.

## Abbreviations

AKI: Acute kidney injury
BMI: Body mass index
BP: Blood pressure
CHW: Community health workers
CV: Cardiovascular
DALY: Disability adjusted life years.
HIV: Human immunodeficiency virus
HRH: Human resources for health
IQR: Interquartile range
LMICs: Low-income and lower middle-income countries
NCDs: Non-communicable diseases
RCTs: Randomized controlled trials.
SSA: Sub-Saharan Africa
UHC: Universal Health Coverage
WHO: World health organization
