# Supplementary material for: Task shifting roles, interventions and outcomes for kidney and cardiovascular health service delivery among African populations: a scoping review
Source: BMC Health Serv Res. 2023 May 5;23:446. doi: 10.1186/s12913-023-09416-5 (PMC10163711; doi:10.1186/s12913-023-09416-5)
Supplement: Supplementary file 1 — Additional file 1: Supplementary Table S1. Search strategy. Supplementary Table S2. Summary of studies aims, interventions, results, and conclusions. Supplementary Table S3. Features of included studies by country. [file 12913_2023_9416_MOESM1_ESM.pdf]

SUPPLEMENTARY MATERIALS:

Supplementary Table S1: Search strategy

**MEDLINE**		
**Search**	**Domain**	**Search terms**
#1	CVD and CV risk factors and kidney diseases	“Hyperlipidemias”[MeSH] OR “hyperlipidemias” OR “hyperlipidemia” OR “hyperlipidaemia” OR “hyperlipidaemias” OR “hyperlipemia” OR “hyperlipemias” OR “hyperlipaemia” OR “hyperlipaemias” OR “lipidemia” OR “lipidaemia” OR “high cholesterol” OR “hypercholesterolemia” OR “hypercholesterolemias” OR “hypercholesteremia” OR “hypercholesteremias” OR “hypercholesterolaemia” OR “hypercholesterolaemias” OR “hypercholesteraemia” OR “dyslipidemia” OR “dyslipidaemia” OR “Diabetes” OR “diabetic” OR "diabetes mellitus"[Mesh] OR “glycemic” OR “glycaemic” OR “obesity” [Mesh] OR “obesity” OR “overweight” OR “obese” OR “proteinuria”[Mesh] OR “proteinuria” OR “albuminuria” OR “hemoglobinuria” OR "Kidney Failure, Chronic"[Mesh] OR “chronic kidney disease” OR “chronic renal disease” OR “chronic renal insufficiency” OR “CKD” OR “end-stage renal disease” OR “chronic kidney failure” OR “chronic renal failure” OR “chronic kidney diseases” OR “chronic renal diseases” OR “chronic renal insufficiencies” OR “end-stage renal diseases” OR “chronic kidney failures” OR “chronic renal failures” OR “renal failure” OR “kidney failure” OR “renal disease” OR “kidney disease” OR “stroke”[Mesh] OR “stroke” OR “strokes” OR “brain vascular accident” OR “brain vascular accidents” OR “apoplexy” OR "cerebrovascular accident” OR "cerebrovascular accidents” OR “cardiomyopathies”[Mesh] OR “cardiomyopathy” OR “cardiomyopathies” OR "myocardial disease” OR "myocardial diseases” OR “myocardiopathy” OR “myocardiopathies” OR “heart neoplasms”[Mesh] OR “heart neoplasm” OR “heart neoplasms” OR "cardiac tumor” OR "cardiac tumors” OR "myocardial tumor” OR "myocardial tumors” OR "cardiac carcinoma” OR "cardiac carcinomas” OR "heart cancer” OR "cardiac cancers” OR "cardiac cancer” OR "heart tumor” OR "heart tumors” OR “myocardial ischemia”[Mesh] OR “myocardial ischemia” OR “myocardial ischemias” OR "ischemic heart disease” OR "ischemic heart diseases” OR “myocardial ischaemia” OR “myocardial ischaemias” OR "ischaemic heart disease” OR "ischaemic heart diseases” OR “acute coronary syndrome” OR “acute coronary syndromes” OR “coronary disease” OR “coronary diseases” OR “coronary artery disease” OR “coronary artery diseases” OR “coronary arteriosclerosis” OR “Coronary atherosclerosis” OR “coronary stenosis” OR “coronary stenoses” OR “coronary restenosis” OR “coronary restenoses” OR “coronary heart disease” OR “coronary heart diseases” OR “coronary thrombosis” OR “coronary thromboses” OR “coronary occlusion” OR “coronary occlusions” OR “myocardial infarction” OR “myocardial infarctions” OR "heart attack” OR “heart attacks” OR “myocardial infarct” OR “myocardial infarcts” OR “heart arrest”[Mesh] OR “heart arrest” OR “heart arrests” OR “cardiac arrest” OR “cardiac arrests” OR “asystole” OR “asystoles” OR “cardiopulmonary arrest” OR “cardiopulmonary arrests” OR “heart failure”[Mesh] OR “heart failure” OR “heart failures” OR “cardiac failure” OR “cardiac failures” OR “myocardial failure” OR “myocardial failures” OR “heart decompensation” OR “hypertensive” OR “blood pressure” OR “hypertension”[Mesh] OR “hypertension” OR “hypertensions” OR “high blood pressure” OR “high blood pressures” OR “cardiovascular diseases”[Mesh] OR “cardiovascular” OR CVD OR “cardiovascular disease” OR “cardiovascular diseases” OR "cardiovascular risk” OR "cardiovascular risks” OR “non communicable” OR “non-communicable” OR NCD OR “salt” OR “tobacco” “physical activity” OR “diet”
#2	Task shifting	((“Task” OR "tasks") AND ("shift" OR "share" OR “sharing” OR "shifted" OR "shifts" OR "shifting")) OR decentralizing OR decentralising OR decentralization OR decentralisation OR (shortage* AND ("physicians"[MeSH] OR “health personnel”[Mesh] OR "physicians" OR "doctors" OR "trained personnel" OR "health workforce" OR “health care workforce" OR “healthcare workforce" OR "health workers" OR "health care workers" OR “healthcare workers" OR "health care providers" OR “health providers" OR “healthcare providers")) OR ("nurse led" OR “nurse-led” OR “nurse-delivery” OR “nurse delivery” OR “nurse-delivered” OR “nurse delivered” OR "primary health care nurse" OR "primary health care nurses" OR "primary health care nursing" OR nurse OR nursing OR pharmacist OR pharmacist-led) OR “non physician” OR “non-physician” OR "nonphysician clinicians" OR "non-physician clinicians" OR “non physician health care workers" OR “nonphysician health care workers" OR “non physician healthcare workers" OR “nonphysician healthcare workers" OR “nonphysician health workers" OR “non physician health workers" OR ("role" AND ("nurse" OR "nurses" OR "nursing")) OR “community health aides”[mesh] OR “community health centers”[mesh] OR “lay health workers" OR “lay health care workers" OR “lay healthcare workers" OR “community health workers" OR “community health care workers" OR “community healthcare workers" OR “community health center” OR “community Health centers” OR “community health centre” OR “community health centres” OR “extended scope practitioner” OR “extended scope practitioners” OR “extended scope practice” OR “enhanced role” OR “role enhancement” OR ((“substitution” OR “substituted” OR “substitute” OR “substituting” OR “substitutes” OR “delegate” OR “delegating” OR “delegates” OR “delegation” OR “delegated”) AND (“physicians”[mesh] OR “physician” OR “physicians” OR “doctor” OR “doctors”))
#3	Africa	Africa OR Angola OR Benin OR Botswana OR Burkina Faso OR Upper Volta OR Burundi OR Urundi OR Cameroon OR Cameroons OR Cape Verde OR Central African Republic OR Chad OR Comoros OR Comoro Islands OR Comores OR Mayotte OR Congo OR Zaire OR Cote d Ivoire OR Ivory Coast OR Democratic Republic of the Congo OR Djibouti OR French Somaliland OR Eritrea OR Ethiopia OR Gabon OR Gabonese Republic OR Gambia OR Ghana OR Gold Coast OR Guinea OR Kenya OR Lesotho OR Basutoland OR Liberia OR Madagascar OR Malagasy Republic OR Malawi OR Nyasaland OR Mali OR Mauritania OR Mauritius OR Mozambique OR Namibia OR Niger OR Nigeria OR Rwanda OR Sao Tome OR Seychelles OR Senegal OR Sierra Leone OR Somalia OR South Africa OR Sudan OR Swaziland OR Tanzania OR Togo OR Togolese Republic OR Uganda OR Zambia OR Zimbabwe OR Rhodesia OR Algeria OR Egypt OR Libya OR Morocco OR Tunisia

#4	Final strategy	#1 AND #2 AND #3

Web of Science

#1	CVD and CV risk factors and kidney diseases	“hyperlipidemias” OR “hyperlipidemia” OR “hyperlipidaemia” OR “hyperlipidaemias” OR “hyperlipemia” OR “hyperlipemias” OR “hyperlipaemia” OR “hyperlipaemias” OR “lipidemia” OR “lipidaemia” OR “high cholesterol” OR “hypercholesterolemia” OR “hypercholesterolemias” OR “hypercholesteremia” OR “hypercholesteremias” OR “hypercholesterolaemia” OR “hypercholesterolaemias” OR “hypercholesteraemia” OR “dyslipidemia” OR “dyslipidaemia” OR “Diabetes” OR “diabetic” OR "diabetes mellitus” OR “glycemic” OR “glycaemic” OR “obesity” OR “obesity” OR “overweight” OR “obese” OR “proteinuria” OR “proteinuria” OR “albuminuria” OR “hemoglobinuria” OR "Kidney Failure, Chronic" OR “chronic kidney disease” OR “chronic renal disease” OR “chronic renal insufficiency” OR “CKD” OR “end-stage renal disease” OR “chronic kidney failure” OR “chronic renal failure” OR “chronic kidney diseases” OR “chronic renal diseases” OR “chronic renal insufficiencies” OR “end-stage renal diseases” OR “chronic kidney failures” OR “chronic renal failures” OR “renal failure” OR “kidney failure” OR “renal disease” OR “kidney disease” OR “stroke” OR “stroke” OR “strokes” OR “brain vascular accident” OR “brain vascular accidents” OR “apoplexy” OR "cerebrovascular accident” OR "cerebrovascular accidents” OR “cardiomyopathies” OR “cardiomyopathy” OR “cardiomyopathies” OR "myocardial disease” OR "myocardial diseases” OR “myocardiopathy” OR “myocardiopathies” OR “heart neoplasms” OR “heart neoplasm” OR “heart neoplasms” OR "cardiac tumor” OR "cardiac tumors” OR "myocardial tumor” OR "myocardial tumors” OR "cardiac carcinoma” OR "cardiac carcinomas” OR "heart cancer” OR "cardiac cancers” OR "cardiac cancer” OR "heart tumor” OR "heart tumors” OR “myocardial ischemia” OR “myocardial ischemia” OR “myocardial ischemias” OR "ischemic heart disease” OR "ischemic heart diseases” OR “myocardial ischaemia” OR “myocardial ischaemias” OR "ischaemic heart disease” OR "ischaemic heart diseases” OR “acute coronary syndrome” OR “acute coronary syndromes” OR “coronary disease” OR “coronary diseases” OR “coronary artery disease” OR “coronary artery diseases” OR “coronary arteriosclerosis” OR “Coronary atherosclerosis” OR “coronary stenosis” OR “coronary stenoses” OR “coronary restenosis” OR “coronary restenoses” OR “coronary heart disease” OR “coronary heart diseases” OR “coronary thrombosis” OR “coronary thromboses” OR “coronary occlusion” OR “coronary occlusions” OR “myocardial infarction” OR “myocardial infarctions” OR “heart attack” OR “heart attacks” OR “myocardial infarct” OR “myocardial infarcts” OR “heart arrest” OR “heart arrest” OR “heart arrests” OR “cardiac arrest” OR “cardiac arrests” OR “asystole” OR “asystoles” OR “cardiopulmonary arrest” OR “cardiopulmonary arrests” OR “heart failure” OR “heart failure” OR “heart failures” OR “cardiac failure” OR “cardiac failures” OR “myocardial failure” OR “myocardial failures” OR “heart decompensation” OR “hypertensive” OR “blood pressure” OR “hypertension”OR “hypertension” OR “hypertensions” OR “high blood pressure” OR “high blood pressures” OR “cardiovascular diseases” OR “cardiovascular” OR CVD OR “cardiovascular disease” OR “cardiovascular diseases” OR "cardiovascular risk” OR "cardiovascular risks” OR “non communicable” OR “non-communicable” OR NCD OR “salt” OR “tobacco” “physical activity” OR “diet”
#2	Task shifting	((“Task” OR "tasks") AND ("shift" OR "share" OR “sharing” OR "shifted" OR "shifts" OR "shifting")) OR decentralizing OR decentralising OR decentralization OR decentralisation OR (shortage* AND ("physicians" OR “health personnel” OR "physicians" OR "doctors" OR "trained personnel" OR "health workforce" OR “health care workforce" OR “healthcare workforce" OR "health workers" OR "health care workers" OR “healthcare workers" OR "health care providers" OR "health providers" OR “healthcare providers")) OR ("nurse led" OR “nurse-led” OR “nurse-delivery” OR “nurse delivery” OR “nurse-delivered” OR “nurse delivered” OR "primary health care nurse" OR "primary health care nurses" OR "primary health care nursing" OR nurse OR nursing OR pharmacist OR pharmacist-led) OR “non physician” OR “non-physician” OR "nonphysician clinicians" OR "non-physician clinicians" OR “non physician health care workers" OR “nonphysician health care workers" OR “non physician healthcare workers" OR “nonphysician healthcare workers" OR “nonphysician health workers" OR “non physician health workers" OR ("role" AND ("nurse" OR "nurses" OR "nursing")) OR “community health aides” OR “community health centers” OR “lay health workers" OR “lay health care workers" OR “lay healthcare workers" OR “community health workers" OR “community health care workers" OR “community healthcare workers" OR “community health center” OR “community Health centers” OR “community health centre” OR “community health centres” OR “extended scope practitioner” OR “extended scope practitioners” OR “extended scope practice” OR “enhanced role” OR “role enhancement” OR ((“substitution” OR “substituted” OR “substitute” OR “substituting” OR “substitutes” OR “delegate” OR “delegating” OR “delegates” OR “delegation” OR “delegated”) AND (“physicians” OR “physician” OR “physicians” OR “doctor” OR “doctors”))
#3	Africa	Africa OR Angola OR Benin OR Botswana OR Burkina Faso OR Upper Volta OR Burundi OR Urundi OR Cameroon OR Cameroons OR Cape Verde OR Central African Republic OR Chad OR Comoros OR Comoro Islands OR Comores OR Mayotte OR Congo OR Zaire OR Cote d Ivoire OR Ivory Coast OR Democratic Republic of the Congo OR Djibouti OR French Somaliland OR Eritrea OR Ethiopia OR Gabon OR Gabonese Republic OR Gambia OR Ghana OR Gold Coast OR Guinea OR Kenya OR Lesotho OR Basutoland OR Liberia OR Madagascar OR Malagasy Republic OR Malawi OR Nyasaland OR Mali OR Mauritania OR Mauritius OR Mozambique OR Namibia OR Niger OR Nigeria OR Rwanda OR Sao Tome OR Seychelles OR Senegal OR Sierra Leone OR Somalia OR South Africa OR Sudan OR Swaziland OR Tanzania OR Togo OR Togolese Republic OR Uganda OR Zambia OR Zimbabwe OR Rhodesia OR Algeria OR Egypt OR Libya OR Morocco OR Tunisia
#4	Final strategy	#1 AND #2 AND #3

EMBASE

#1	CVD and CV risk factors and kidney diseases	‘hyperlipidemias’ OR ‘hyperlipidemia’ OR ‘hyperlipidaemia’ OR ‘hyperlipidaemias’ OR ‘hyperlipemia’ OR ‘hyperlipemias’ OR ‘hyperlipaemia’ OR ‘hyperlipaemias’ OR ‘lipidemia’ OR ‘lipidaemia’ OR ‘high cholesterol’ OR ‘hypercholesterolemia’ OR ‘hypercholesterolemias’ OR ‘hypercholesteremia’ OR ‘hypercholesteremias’ OR ‘hypercholesterolaemia’ OR ‘hypercholesterolaemias’ OR ‘hypercholesteraemia’ OR ‘dyslipidemia’ OR ‘dyslipidaemia’ OR ‘Diabetes’ OR ‘diabetic’ OR ‘diabetes mellitus’

		OR ‘glycemic’ OR ‘glycaemic’ OR ‘obesity’ OR ‘obesity’ OR ‘overweight’ OR ‘obese’ OR ‘proteinuria’ OR ‘proteinuria’ OR ‘albuminuria’ OR ‘hemoglobinuria’ OR ‘Kidney Failure, Chronic’ OR ‘chronic kidney disease’ OR ‘chronic renal disease’ OR ‘chronic renal insufficiency’ OR ‘CKD’ OR ‘end-stage renal disease’ OR ‘chronic kidney failure’ OR ‘chronic renal failure’ OR ‘chronic kidney diseases’ OR ‘chronic renal diseases’ OR ‘chronic renal insufficiencies’ OR ‘end-stage renal diseases’ OR ‘chronic kidney failures’ OR ‘chronic renal failures’ OR ‘renal failure’ OR ‘kidney failure’ OR ‘renal disease’ OR ‘kidney disease’ OR ‘stroke’ OR ‘stroke’ OR ‘strokes’ OR ‘brain vascular accident’ OR ‘brain vascular accidents’ OR ‘apoplexy’ OR ‘cerebrovascular accident’ OR ‘cerebrovascular accidents’ OR ‘cardiomyopathies’ OR ‘cardiomyopathy’ OR ‘cardiomyopathies’ OR ‘myocardial disease’ OR ‘myocardial diseases’ OR ‘myocardiopathy’ OR ‘myocardiopathies’ OR ‘heart neoplasms’ OR ‘heart neoplasm’ OR ‘heart neoplasms’ OR ‘cardiac tumor’ OR ‘cardiac tumors’ OR ‘myocardial tumor’ OR ‘myocardial tumors’ OR ‘cardiac carcinoma’ OR ‘cardiac carcinomas’ OR ‘heart cancer’ OR ‘cardiac cancers’ OR ‘cardiac cancer’ OR ‘heart tumor’ OR ‘heart tumors’ OR ‘myocardial ischemia’ OR ‘myocardial ischemia’ OR ‘myocardial ischemias’ OR ‘ischemic heart disease’ OR ‘ischemic heart diseases’ OR ‘myocardial ischaemia’ OR ‘myocardial ischaemias’ OR ‘ischaemic heart disease’ OR ‘ischaemic heart diseases’ OR ‘acute coronary syndrome’ OR ‘acute coronary syndromes’ OR ‘coronary disease’ OR ‘coronary diseases’ OR ‘coronary artery disease’ OR ‘coronary artery diseases’ OR ‘coronary arteriosclerosis’ OR ‘Coronary atherosclerosis’ OR ‘coronary stenosis’ OR ‘coronary stenoses’ OR ‘coronary restenosis’ OR ‘coronary restenoses’ OR ‘coronary heart disease’ OR ‘coronary heart diseases’ OR ‘coronary thrombosis’ OR ‘coronary thromboses’ OR ‘coronary occlusion’ OR ‘coronary occlusions’ OR ‘myocardial infarction’ OR ‘myocardial infarctions’ OR ‘heart attack’ OR ‘heart attacks’ OR ‘myocardial infarct’ OR ‘myocardial infarcts’ OR ‘heart arrest’ OR ‘heart arrest’ OR ‘heart arrests’ OR ‘cardiac arrest’ OR ‘cardiac arrests’ OR ‘asystole’ OR ‘asystoles’ OR ‘cardiopulmonary arrest’ OR ‘cardiopulmonary arrests’ OR ‘heart failure’ OR ‘heart failure’ OR ‘heart failures’ OR ‘cardiac failure’ OR ‘cardiac failures’ OR ‘myocardial failure’ OR ‘myocardial failures’ OR ‘heart decompensation’ OR ‘hypertensive’ OR ‘blood pressure’ OR ‘hypertension’ OR ‘hypertension’ OR ‘hypertensions’ OR ‘high blood pressure’ OR ‘high blood pressures’ OR ‘cardiovascular diseases’ OR ‘cardiovascular’ OR CVD OR ‘cardiovascular disease’ OR ‘cardiovascular diseases’ OR ‘cardiovascular risk’ OR ‘cardiovascular risks’ OR ‘non communicable’ OR ‘non-communicable’ OR NCD OR ‘salt’ OR ‘tobacco’ ‘physical activity’ OR ‘diet’
#2	Task shifting	((‘Task’ OR ‘tasks’) AND (‘shift’ OR ‘share’ OR ‘sharing’ OR ‘shifted’ OR ‘shifts’ OR ‘shifting’)) OR decentralizing OR decentralising OR decentralization OR decentralisation OR (shortage* AND (‘physicians’ OR ‘health personnel’ OR ‘physicians’ OR ‘doctors’ OR ‘trained personnel’ OR ‘health workforce’ OR ‘health care workforce’ OR ‘healthcare workforce’ OR ‘health workers’ OR ‘health care workers’ OR ‘healthcare workers’ OR ‘health care providers’ OR ‘health providers’ OR ‘healthcare providers’)) OR (‘nurse led’ OR ‘nurse-led’ OR ‘nurse-delivery’ OR ‘nurse delivery’ OR ‘nurse-delivered’ OR ‘nurse delivered’ OR ‘primary health care nurse’ OR ‘primary health care nurses’ OR ‘primary health care nursing’ OR nurse OR nursing OR pharmacist OR pharmacist-led) OR ‘non physician’ OR ‘non-physician’ OR ‘nonphysician clinicians’ OR ‘non-physician clinicians’ OR ‘non physician health care workers’ OR ‘nonphysician health care workers’ OR ‘non physician healthcare workers’ OR ‘nonphysician healthcare workers’ OR ‘nonphysician health workers’ OR ‘non physician health workers’ OR (‘role’ AND (‘nurse’ OR ‘nurses’ OR ‘nursing’)) OR ‘community health aides’ OR ‘community health centers’ OR ‘lay health workers’ OR ‘lay health care workers’ OR ‘lay healthcare workers’ OR ‘community health workers’ OR ‘community health care workers’ OR ‘community healthcare workers’ OR ‘community health center’ OR ‘community Health centers’ OR ‘community health centre’ OR ‘community health centres’ OR ‘extended scope practitioner’ OR ‘extended scope practitioners’ OR ‘extended scope practice’ OR ‘enhanced role’ OR ‘role enhancement’ OR ((‘substitution’ OR ‘substituted’ OR ‘substitute’ OR ‘substituting’ OR ‘substitutes’ OR ‘delegate’ OR ‘delegating’ OR ‘delegates’ OR ‘delegation’ OR ‘delegated’) AND (‘physicians’ OR ‘physician’ OR ‘physicians’ OR ‘doctor’ OR ‘doctors’))
#3	Africa	Africa OR Angola OR Benin OR Botswana OR Burkina Faso OR Upper Volta OR Burundi OR Urundi OR Cameroon OR Cameroons OR Cape Verde OR Central African Republic OR Chad OR Comoros OR Comoro Islands OR Comores OR Mayotte OR Congo OR Zaire OR Cote d Ivoire OR Ivory Coast OR Democratic Republic of the Congo OR Djibouti OR French Somaliland OR Eritrea OR Ethiopia OR Gabon OR Gabonese Republic OR Gambia OR Ghana OR Gold Coast OR Guinea OR Kenya OR Lesotho OR Basutoland OR Liberia OR Madagascar OR Malagasy Republic OR Malawi OR Nyasaland OR Mali OR Mauritania OR Mauritius OR Mozambique OR Namibia OR Niger OR Nigeria OR Rwanda OR Sao Tome OR Seychelles OR Senegal OR Sierra Leone OR Somalia OR South Africa OR Sudan OR Swaziland OR Tanzania OR Togo OR Togolese Republic OR Uganda OR Zambia OR Zimbabwe OR Rhodesia OR Algeria OR Egypt OR Libya OR Morocco OR Tunisia
#4	Final strategy	#1 AND #2 AND #3

CINAHL

#1	CVD and CV risk factors and kidney diseases	‘hyperlipidemias’ OR ‘hyperlipidemia’ OR ‘hyperlipidaemia’ OR ‘hyperlipidaemias’ OR ‘hyperlipemia’ OR ‘hyperlipemias’ OR ‘hyperlipaemia’ OR ‘hyperlipaemias’ OR ‘lipidemia’ OR ‘lipidaemia’ OR ‘high cholesterol’ OR ‘hypercholesterolemia’ OR ‘hypercholesterolemias’ OR ‘hypercholesteremia’ OR ‘hypercholesteremias’ OR ‘hypercholesterolaemia’ OR ‘hypercholesterolaemias’ OR ‘hypercholesteraemia’ OR ‘dyslipidemia’ OR ‘dyslipidaemia’ OR ‘Diabetes’ OR ‘diabetic’ OR ‘diabetes mellitus’ OR ‘glycemic’ OR ‘glycaemic’ OR ‘obesity’ OR ‘obesity’ OR ‘overweight’ OR ‘obese’ OR ‘proteinuria’ OR ‘proteinuria’ OR ‘albuminuria’ OR ‘hemoglobinuria’ OR ‘Kidney Failure, Chronic’ OR ‘chronic kidney disease’ OR ‘chronic renal disease’ OR ‘chronic renal insufficiency’ OR ‘CKD’ OR ‘end-stage renal disease’ OR ‘chronic kidney failure’ OR ‘chronic renal failure’ OR ‘chronic kidney diseases’ OR ‘chronic renal diseases’ OR ‘chronic renal insufficiencies’ OR ‘end-stage renal diseases’ OR ‘chronic kidney failures’ OR ‘chronic renal failures’ OR ‘renal failure’ OR ‘kidney failure’ OR ‘renal disease’ OR ‘kidney disease’ OR ‘stroke’ OR ‘stroke’ OR ‘strokes’ OR ‘brain vascular accident’ OR ‘brain vascular accidents’ OR ‘apoplexy’ OR ‘cerebrovascular accident’ OR ‘cerebrovascular accidents’ OR ‘cardiomyopathies’ OR ‘cardiomyopathy’ OR ‘cardiomyopathies’ OR ‘myocardial disease’ OR ‘myocardial diseases’ OR ‘myocardiopathy’ OR ‘myocardiopathies’ OR ‘heart neoplasms’ OR ‘heart neoplasm’ OR ‘heart neoplasms’ OR ‘cardiac tumor’ OR ‘cardiac tumors’ OR ‘myocardial tumor’ OR ‘myocardial tumors’ OR ‘cardiac carcinoma’ OR ‘cardiac carcinomas’ OR ‘heart cancer’ OR

		‘cardiac cancers’ OR ‘cardiac cancer’ OR ‘heart tumor’ OR ‘heart tumors’ OR ‘myocardial ischemia’ OR ‘myocardial ischemia’ OR ‘myocardial ischemias’ OR ‘ischemic heart disease’ OR ‘ischemic heart diseases’ OR ‘myocardial ischaemia’ OR ‘myocardial ischaemias’ OR ‘ischaemic heart disease’ OR ‘ischaemic heart diseases’ OR ‘acute coronary syndrome’ OR ‘acute coronary syndromes’ OR ‘coronary disease’ OR ‘coronary diseases’ OR ‘coronary artery disease’ OR ‘coronary artery diseases’ OR ‘coronary arteriosclerosis’ OR ‘Coronary atherosclerosis’ OR ‘coronary stenosis’ OR ‘coronary stenoses’ OR ‘coronary restenosis’ OR ‘coronary restenoses’ OR ‘coronary heart disease’ OR ‘coronary heart diseases’ OR ‘coronary thrombosis’ OR ‘coronary thromboses’ OR ‘coronary occlusion’ OR ‘coronary occlusions’ OR ‘myocardial infarction’ OR ‘myocardial infarctions’ OR ‘heart attack’ OR ‘heart attacks’ OR ‘myocardial infarct’ OR ‘myocardial infarcts’ OR ‘heart arrest’ OR ‘heart arrest’ OR ‘heart arrests’ OR ‘cardiac arrest’ OR ‘cardiac arrests’ OR ‘asystole’ OR ‘asystoles’ OR ‘cardiopulmonary arrest’ OR ‘cardiopulmonary arrests’ OR ‘heart failure’ OR ‘heart failure’ OR ‘heart failures’ OR ‘cardiac failure’ OR ‘cardiac failures’ OR ‘myocardial failure’ OR ‘myocardial failures’ OR ‘heart decompensation’ OR ‘hypertensive’ OR ‘blood pressure’ OR ‘hypertension’ OR ‘hypertension’ OR ‘hypertensions’ OR ‘high blood pressure’ OR ‘high blood pressures’ OR ‘cardiovascular diseases’ OR ‘cardiovascular’ OR CVD OR ‘cardiovascular disease’ OR ‘cardiovascular diseases’ OR ‘cardiovascular risk’ OR ‘cardiovascular risks’ OR ‘non communicable’ OR ‘non-communicable’ OR NCD OR ‘salt’ OR ‘tobacco’ ‘physical activity’ OR ‘diet’
#2	Task shifting	((‘Task’ OR ‘tasks’) AND (‘shift’ OR ‘share’ OR ‘sharing’ OR ‘shifted’ OR ‘shifts’ OR ‘shifting’)) OR decentralizing OR decentralising OR decentralization OR decentralisation OR (shortage* AND (‘physicians’ OR ‘health personnel’ OR ‘physicians’ OR ‘doctors’ OR ‘trained personnel’ OR ‘health workforce’ OR ‘health care workforce’ OR ‘healthcare workforce’ OR ‘health workers’ OR ‘health care workers’ OR ‘healthcare workers’ OR ‘health care providers’ OR ‘health providers’ OR ‘healthcare providers’)) OR (‘nurse led’ OR ‘nurse-led’ OR ‘nurse-delivery’ OR ‘nurse delivery’ OR ‘nurse-delivered’ OR ‘nurse delivered’ OR ‘primary health care nurse’ OR ‘primary health care nurses’ OR ‘primary health care nursing’ OR nurse OR nursing OR pharmacist OR pharmacist-led) OR ‘non physician’ OR ‘non-physician’ OR ‘nonphysician clinicians’ OR ‘non-physician clinicians’ OR ‘non physician health care workers’ OR ‘nonphysician health care workers’ OR ‘non physician healthcare workers’ OR ‘nonphysician healthcare workers’ OR ‘nonphysician health workers’ OR ‘non physician health workers’ OR (‘role’ AND (‘nurse’ OR ‘nurses’ OR ‘nursing’)) OR ‘community health aides’ OR ‘community health centers’ OR ‘lay health workers’ OR ‘lay health care workers’ OR ‘lay healthcare workers’ OR ‘community health workers’ OR ‘community health care workers’ OR ‘community healthcare workers’ OR ‘community health center’ OR ‘community Health centers’ OR ‘community health centre’ OR ‘community health centres’ OR ‘extended scope practitioner’ OR ‘extended scope practitioners’ OR ‘extended scope practice’ OR ‘enhanced role’ OR ‘role enhancement’ OR ((‘substitution’ OR ‘substituted’ OR ‘substitute’ OR ‘substituting’ OR ‘substitutes’ OR ‘delegate’ OR ‘delegating’ OR ‘delegates’ OR ‘delegation’ OR ‘delegated’) AND (‘physicians’ OR ‘physician’ OR ‘physicians’ OR ‘doctor’ OR ‘doctors’))
#3	Africa	Africa OR Angola OR Benin OR Botswana OR Burkina Faso OR Upper Volta OR Burundi OR Urundi OR Cameroon OR Cameroons OR Cape Verde OR Central African Republic OR Chad OR Comoros OR Comoro Islands OR Comores OR Mayotte OR Congo OR Zaire OR Cote d Ivoire OR Ivory Coast OR Democratic Republic of the Congo OR Djibouti OR French Somaliland OR Eritrea OR Ethiopia OR Gabon OR Gabonese Republic OR Gambia OR Ghana OR Gold Coast OR Guinea OR Kenya OR Lesotho OR Basutoland OR Liberia OR Madagascar OR Malagasy Republic OR Malawi OR Nyasaland OR Mali OR Mauritania OR Mauritius OR Mozambique OR Namibia OR Niger OR Nigeria OR Rwanda OR Sao Tome OR Seychelles OR Senegal OR Sierra Leone OR Somalia OR South Africa OR Sudan OR Swaziland OR Tanzania OR Togo OR Togolese Republic OR Uganda OR Zambia OR Zimbabwe OR Rhodesia OR Algeria OR Egypt OR Libya OR Morocco OR Tunisia
#4	Final strategy	#1 AND #2 AND #3

Supplementary Table S2: Features of included studies by country.

Variables	Overall	South Africa	Nigeria	Ghana	Kenya	Cameroon	DRC	Ethiopia	Malawi	Uganda	Rwanda
**Number of studies (%)**	33 (100)	10 (30.3)	7 (21.2)	4 (12.1)	4 (12.1)	3 (9.1)	1 (3.0)	1 (3.0)	1 (3.0)	1 (3.0)	1 (3.0)
**Sample size**	38,396	21,108	2,449	2,326	5,992	1,475	260	220	104	4,300	162
**Female (%)**	65	73.4	53.9	64.7	61.5	47.4	53.1	35.2	51.9	78	79.6
**Study design [n (%)]**											
- RCT	6 (18.2)	3 (50.0)	2 (33.3)	1 (16.7)	0 (0.0)	0 (0.0)	0 (0.0)	0 (0.0)	0 (0.0)	0 (0.0)	0 (0.0)
- Prospective	19 (57.6)	7 (36.8)	3 (15.8)	3 (15.8)	0 (0.0)	3 (15.8)	0 (0.0)	1 (5.3)	1 (5.3)	0 (0.0)	1 (5.3)
- Cross-sectional	8 (24.2)	0 (0.0)	2 (25.0)	0 (0.0)	4 (50.0)	0 (0.0)	1 (12.5)	0 (0.0)	0 (0.0)	1 (12.5)	0 (0.0)
**Study setting [n (%)]**											
- Rural	8 (24.2)	5 (62.5)	0 (0.0)	0 (0.0)	1 (12.5)	1 (12.5)	0 (0.0)	0 (0.0)	0 (0.0)	1 (12.5)	0 (0.0)
- Urban	12 (36.4)	1 (8.3)	6 (50.0)	2 (16.7)	1 (8.3)	0 (0.0)	1 (8.3)	0 (0.0)	1 (8.3)	0 (0.0)	0 (0.0)
- Rural + Urban	13 (39.4)	4 (30.8)	1 (7.7)	2 (15.4)	2 (15.4)	2 (15.4)	0 (0.0)	1 (7.7)	0 (0.0)	0 (0.0)	1 (7.7)
**Population studied [n (%)]**											
- Hypertension only	16 (48.5)	2 (12.5)	5 (31.3)	3 (18.8)	2 (12.5)	1 (6.3)	1 (6.3)	0 (0.0)	0 (0.0)	1 (6.3)	1 (6.3)
- Diabetes mellitus only	5 (15.2)	4 (80.0)	0 (0.0)	0 (0.0)	0 (0.0)	0 (0.0)	0 (0.0)	1 (20.0)	0 (0.0)	0 (0.0)	0 (0.0)
- Kidney disease (AKI)	1 (3.0)	0 (0.0)	0 (0.0)	0 (0.0)	0 (0.0)	0 (0.0)	0 (0.0)	0 (0.0)	1 (100.0)	0 (0.0)	0 (0.0)
- Multiple risk factors	11 (33.3)	4 (36.4)	2 (18.2)	1 (9.1)	2 (18.2)	2 (18.2)	0 (0.0)	0 (0.0)	0 (0.0)	0 (0.0)	0 (0.0)
**TS to health worker [n (%)]:**											
- Nurses	19 (57.6)	6 (31.6)	2 (10.5)	3 (15.8)	2 (10.5)	2 (10.5)	1 (5.3)	1 (5.3)	1 (5.3)	0 (0.0)	1 (5.3)
- Pharmacists	6 (18.2)	1 (16.7)	4 (66.7)	1 (16.7)	0 (0.0)	0 (0.0)	0 (0.0)	0 (0.0)	0 (0.0)	0 (0.0)	0 (0.0)
- CHW	5 (15.2)	2 (40.0)	1 (20.0)	0 (0.0)	2 (40.0)	0 (0.0)	0 (0.0)	0 (0.0)	0 (0.0)	0 (0.0)	0 (0.0)
- Others**	3 (9.1)	1 (33.3)	0 (0.0)	0 (0.0)	0 (0.0)	1 (33.3)	0 (0.0)	0 (0.0)	0 (0.0)	1 (33.3)	0 (0.0)
**TS role [n (%)]**											
- Triage	13 (39.4)	5 (38.5)	3 (23.1)	1 (7.7)	3 (23.1)	0 (0.0)	0 (0.0)	0 (0.0)	0 (0.0)	1 (7.7)	0 (0.0)
- Education / counselling	24 (72.7)	8 (33.3)	6 (25.0)	4 (16.7)	0 (0.0)	3 (12.5)	0 (0.0)	1 (4.2)	1 (4.2)	0 (0.0)	1 (4.2)
- Disease screening / detection	24 (72.7)	7 (29.2)	4 (16.7)	3 (12.5)	3 (12.5)	3 (12.5)	0 (0.0)	1 (4.2)	1 (4.2)	1 (4.2)	1 (4.2)
- Management / Adherence	28 (84.9)	10 (35.7)	6 (21.4)	3 (10.7)	2 (7.1)	3 (10.7)	1 (3.6)	1 (3.6)	1 (3.6)	0 (0.0)	1 (3.6)

**Others – health promoters, medicine counter assistant, non-physician clinician
Abbreviations: RCT – randomized control studies; TS – task shifting; DRC – Democratic Republic of Congo; CHW – community health workers

Supplementary Table S3: Summary of studies aims, interventions, results, and conclusions.

First author [Ref]	Publication year	Description of intervention	Summary of study aims	Summary of study results	Summary of study conclusions
Coleman et al [24]	1998	Nurses provided primary care and assessed medication adherence for various NCDs.	To assess the design and implementation of a nurse-led NCD service based on clinical protocols in a resource-poor area of South Africa.	The protocols enabled the nurses to control the clinical condition of 68% of patients with hypertension, 82% of those with non-insulin-dependent diabetes, and 84% of those with asthma.	The use of simple protocols and treatment strategies that were responsive to the local situation enabled the majority of patients to receive convenient and appropriate management of their NCD led by Nurses at their local primary care facility.
Gill et al [25]	2008	Nurses performed diabetes diagnosis, treatment, and care	To evaluate a nurse-led protocol and an education-based methodology.	Education alone, without any changes in drug type or dose, improved HbA1c control.	They concluded that in rural Africa, a simple protocol and education-based diabetes care system could be successfully implemented and managed by nurses.
Price et al [26]	2011	Nurses trained to use an algorithm to manage diabetes.	To determine the long-term (4 years) glycaemic outcome of a structured nurse-led intervention programme for T2DM	HbA1c fell significantly to 8.1±2.2% at 6 months and 7.5±2.0% at 18 months. By 24 months, it had risen (8.4±2.3%), and at 4 years post-intervention it was 9.7±4.0% (still significantly lower than baseline, P = 0.015).	The intervention led to marked HbA1c improvements up to 18 months follow-up, but thereafter there was 'glycaemic slippage'.
Mash et al [27]	2014	A health promoter-led educational intervention in rural communities	To evaluate the effectiveness of group education, led by health promoters using a guiding style, for people with T2DM	A significant reduction in mean systolic (-4.65 mmHg, 95% CI 9.18 to -0.12; P = 0.04) and diastolic blood pressure (-3.30 mmHg, 95% CI -5.35 to -1.26; P = 0.002).	The reduction in participants' mean blood pressure is likely to be of clinical significance.
Muchiri et al [28]	2015	Community health workers performed nutritional education for diabetes patients.	To evaluate the effect of a participant-customised nutrition education programme on glycated Hb (HbA1c), blood lipids, blood pressure, BMI, and dietary behaviours in patients with T2DM	Differences in HbA1c (primary outcome) were -0•64 % (P=0•15) at 6 months and -0•63 % (P=0•16) at 12 months in favour of the intervention group).	Nutrition education improved specific dietary behaviours. Group education and hands-on activities appeared to contribute to the improvement.
Fairall et al [29]	2016	Primary care nurses' capacity to manage NCDs.	To evaluate the effectiveness of nurse-based intervention, which combines provision of an integrated management tool with educational outreach.	Treatment intensification rates in intervention clinics were not superior to those in the control clinics (hypertension: 44% in the intervention group versus 40% in the control group, risk ratio [RR] 1.08 [95% CI 0.94 to 1.24; *p* = 0.252]; diabetes: 57% versus 50%, RR 1.10 [0.97 to 1.24; *p* = 0.126]; chronic respiratory disease: 14% versus 12%, RR 1.08 [0.75 to 1.55; *p* = 0.674]), nor was case detection of depression (18% versus 24%, RR 0.76 [0.53 to 1.10; *p* = 0.142]). No adverse effects of the nurses' expanded scope of practice were observed.	Educational outreach to primary care nurses to train them in the use of a management tool involving an expanded role in managing NCDs was feasible and safe.
Morris-Paxton et al [30]	2018	Community health workers intervention for diagnosis and treatment for T2DM and hypertension.	To report on the follow-up of patients in the community with known hypertension or diabetes or who were deemed at-risk (as identified during a prior community-wide survey).	Of the 1885 participants followed up by the community health outreach workers, 1702 were known to be hypertensive and 183 were deemed at-risk [of these, only 24 (13.2%) had normal or high normal systolic BP readings]. There were 341 participants with diabetes and 34 at-risk of diabetes [of these, 28 (82%) had levels of 11 mmol/l or higher at follow-up]. There was a significant improvement in BP and glucose control over repeated visits.	The follow-up of patients with hypertension or diabetes as well as those individuals at-risk adds value to hypertension and glucose control.
Rampamba et al [31]	2019	Pharmacist-led patient counseling and education model	To evaluate the impact of a pharmacist-led patient counseling and education model to empower hypertensive patients on chronic medication.	An improved understanding of what normal BP is in the intervention group compared to the control group (P < 0.001), whereas a 9.1% improvement was also observed in the intervention group	A pharmacist-led patient counseling and education model can help improve patients' hypertension knowledge and BP control. These should increasingly become routine, aiming to improve chronic disease management.
Madela et al [32]	2020	CHW intervention in diabetes and hypertension care	To describe the implementation of a community-based programme, which equipped a CHWs to perform screening and clinical tasks	More than one in five (21.0%, *n* = 1448), of those with no previous hypertension diagnosis, were found to have raised blood pressure at screening, representing newly detected cases.	Community caregivers played an important role in early detection of raised blood pressure and raised blood glucose, and in referring patients to primary care.
Sharp et al [33]	2022	Decentralized nurse-led NCD care clinics	The objective of this study is to assess the feasibility and impact of decentralised care for NCDs within nurse-led clinics, in order improve access and inform healthcare planning in Eswatini and similar settings.	One thousand one hundred twenty-five patients were recruited to the study. Of these patients, 573 attended for at least 4 appointments. There was a significant reduction in mean BP among hypertensive patients after four visits of 9.9 mmHg systolic and 4.7 mmHg diastolic (p = 0.01), and a	The findings suggest that management of diabetes and hypertension care in a rural district setting can be safely delivered by nurses in community clinics according to a shared care protocol. Improved access is likely to lead to improved patient compliance with treatment.

				non-significant reduction in fasting blood glucose among diabetic patients of 1.2 mmol/l (p=0.2)	
Oparah et al [34]	2006	Pharmacist-led counselling and reinforcement of adherence to medications in a cohort of hypertensive adults	To describe pharmaceutical care interventions provided to hypertensive patients in a Nigerian community pharmacy setting, and to assess the impact of the practice on selected patient outcomes	There was a significant difference between mean SBP at baseline (187.67 ± 29.46 mmHg) and at the end of the study (137.22 ± 21.65 mmHg), P < 0.0001. Changes in mean DBP at baseline (117.56 ± 21.65) and end of study (89 ± 17.23), were also significant (P < 0.0001). Some 27 (75%) reached SBP goals while 25 (69%) attained DBP goals. Prior to the pharmaceutical care intervention, 27 (75%) were not aware that salt intake was a risk factor in hypertension, and a similar proportion thought that hypertension was curable.	Pharmaceutical care provided to hypertensive patients in a Nigerian community pharmacy setting improved blood pressure control and overall patient satisfaction with pharmaceutical service
Adeyemo et al [35]	2013	Monitoring of adherence to prescribed anti-hypertensive medications by Nurses.	The primary goal of this study was to expand the evidence base necessary to guide hypertension treatment and control programs in Africa.	Among participants who completed the 6-month trial, overall adherence was high (~77% of participants took > 98% of prescribed pills). Adherence did not differ by treatment arm but was better at the rural than the urban site and among those with higher baseline BP. Hypertension control (BP < 140/90 mmHg) was achieved in ~66% of participants at 6 months.	This community-based intervention confirms relatively modest default rates compared to industrialized societies and suggests that medication adherence can be high in developing world settings in clinic attenders.
Nelissen et al [36]	2018	Pharmacists-led hypertension medication treatment and counselling with remote instructions from cardiologists	To assess the feasibility of pharmacist-based hypertension care that includes remote patient monitoring by cardiologists through mHealth	Mean systolic blood pressure decreased 9.9 mmHg (SD: 18). Blood pressure on target increased from 24 to 56% and an additional 10% had an improved blood pressure at endline, however this was not associated with duration of mHealth activity. Patients were satisfied because of accessibility, attention, adherence, and information provision.	Most patients were satisfied, and their mean blood pressure significantly reduced. Usage of the mHealth application, pharmacy incentives, and a modified financing model are opportunities for improvement.
Ozoememna et al [37]	2019	Nurse-led educational interventions to improve hypertension knowledge	This study aimed to determine the effectiveness of a health education intervention in improving hypertension knowledge, prevention, and self-care practices among retirees.	The paired comparison analysis showed that the mean HTN knowledge score significantly increased in the T-group between baseline and 1 month (4th month) post-intervention compared to those in the C-group (P <0.0001). Also, medication adherence (P = 0.000) improved significantly in the T-group compared to the control between baseline and 1 month after intervention.	Community-based health education intervention targeted at older adults can increase hypertension knowledge, improve prevention and self-care practices of hypertension at the population level
Amadi et al [38]	2020	Adults were screened in community pharmacies for these factors	To study the effectiveness of opportunistic screening for CVD risk factors in community pharmacies on apparently healthy individuals and also to identify perceived barriers.	Prevalence of hypertension was 28.2% in all subjects, 30.9% in males and 26.3% in females, p < 0.001. Using BP > 130/80 mmHg prevalence of hypertension was 55.1%. Diabetes was detected in 3% of the subjects while 45.3% had hypercholesterolaemia. In total, 64.1% of the subjects were diagnosed with CVD risk factors for the first time	Opportunistic screening for CVD risk factors is possible in community pharmacies and has the ability to detect previously undiagnosed risk factors. This community pharmacy-based model could serve as a cost-effective approach to primary prevention of CVD.
Ojji et al [39]	2020	Community health workers support for hypertension treatment and monitoring.	The aim was to evaluate the feasibility and effect of community health worker support and self-home BP monitoring compared with usual care on BP treatment	At the four-week follow up, the mean SBP differences were –31 (12), –27 (14) and –21 (8) mmHg in the community health worker-supported, home blood pressure-monitoring and usual care groups, respectively (p = 0.02). There were no differences in DBP at the four-week follow up	The study demonstrates that community health worker support and home blood pressure monitoring are feasible and may be effective in primary care settings in Nigeria
Onyinye et al [40]	2021	Pharmacists-led educational intervention	The objective of this study was to evaluate the effect of a pharmacist-led intervention on self-management practices among hypertensive-diabetic patients receiving care in a Nigeria tertiary hospital	It can was observed that the intervention group differed positively from the endpoint in medication adherence (p<0.001); physical activity (p<0.001); weight management practices (p<0.0010, and alcohol consumption (p<0.001).	A pharmacist led educational intervention had a positive impact on the self-management practices of hypertensive-diabetes patients.
Sarfo et al [41]	2015	Nurse-guided hypertension control using mHealth among stroke patients.	To test whether an mHealth technology-enabled, nurse-led, multi-level integrated approach is effective in improving BP control among Ghanaian stroke patients	At month 9, proportion on the intervention versus controls with BP < 140/90 mmHg was 14/30 (46.7%) versus 12/30 (40.0%), p = 0.79 by intention-to-treat; systolic BP < 140 mmHg was 22/30 (73.3%) versus 13/30 (43.3%), p = 0.035. Mean ± SD medication possession ratio was 0.95 ± 0.16 on intervention versus 0.98 ± 0.24 in the control arm, p = 0.56.	The study demonstrated feasibility and signal of improvement in BP control among stroke survivors in a resource-limited setting via an mHealth intervention that required task shifting.
Marfo et al [42]	2016	Pharmacist-led hypertension preventative service	The objective of the study was to explore the feasibility and acceptability of a pharmacist-led hypertension preventative service in the community pharmacy.	34 participants who were referred to the physician by the pharmacists, 10 (29%) were diagnosed with hypertension and an antihypertensive was prescribed. Some risk factors for hypertension where also identified by the pharmacists	The findings suggest that pharmacists can detect hypertension and promote some lifestyle changes among clients with pre-hypertension

Ogedegbe et al [43]	2018	Nurse-led service for hypertension control	To evaluate the comparative effectiveness of provision of health insurance coverage (HIC) alone versus a nurse-led task shifting strategy for hypertension control (TASSH) plus HIC on systolic blood pressure reduction among patients with uncontrolled hypertension in Ghana	In intention-to-treat analyses adjusted for clustering, the TASSH + HIC group had a greater SBP reduction (−20.4 mm Hg; 95% CI −25.2 to −15.6) than the HIC group (−16.8 mm Hg; 95% CI −19.2 to −15.6), with a statistically significant between-group difference of −3.6 mm Hg (95% CI −6.1 to −0.5; *p* = 0.021). Blood pressure control improved significantly in both groups (55.2%, 95% CI 50.0% to 60.3%, for the TASSH + HIC group versus 49.9%, 95% CI 44.9% to 54.9%, for the HIC group), with a non-significant between group difference of 5.2% (95% CI −1.8% to 12.4%; *p* = 0.29).	Provision of health insurance coverage plus a nurse-led task shifting strategy was associated with a greater reduction in SBP than provision of health insurance coverage alone, among patients with uncontrolled hypertension in Ghana
Adler et al [44]	2019	Nurse-led community program for hypertension care	To evaluate the effectiveness of the Community-based Hypertension Improvement Project	After 1 year of intervention, 72% (95% CI: 67% to 77%) of participants had their hypertension under control. Systolic BP was reduced by 12.2 mm Hg (95% CI: 14.4 to 10.1) and diastolic BP by 7.5 mmHg (95% CI: 9.9 to 6.1)	Patients retained in the program had increased BP control. However, high loss to follow-up limits potential public health impact of these types of programmes.
Some et al. [45]	2016	Nurse-led NCD treatment program	To evaluate adherence to clinical decision support protocols when the care of five NCDs (HTN, T2DM, asthma, epilepsy and sickle cell disease) was shifted from clinical officers to nurses	There were 3,554 consultations (2025 patients); 733 (21%) were by nurses out of which 725 met the inclusion criteria among 616 patients. Hypertension (64%, 397/616) was the most frequent NCD followed by asthma (17%, 106/616) and diabetes mellitus (15%, 95/616). Adherence to screening questions ranged from 65% to 86%, with an average of 69%. BP measurements were completed in 89% and 96% of those required. Only 17 (2%) consultations were referred back to clinical officers.	Nurses are able to adhere to protocols for managing stable NCD patients based on clear and standardized protocols and guidelines, thus paving the way towards task shifting of NCD care to nurses to help relieve the significant healthcare gap in developing countries
Mannik et al [46]	2018	Use of a nurse-run, mobile health app to screen for CVD risk factors in rural Kenya	To design and test a novel mobile health (mHealth) tool for use by community health workers (CHWs) to identify individuals at high CVD risk who would benefit from education and/or pharmacologic interventions	Five CHWs screened 2865 subjects in remote rural communities in Kenya over a 22-month period (2015–2017). The median age of subjects was 50 (interquartile range 43–60) and 1581 (55%) were female. The point prevalence of hypertension (systolic blood pressure > 140 mmHg), diabetes and tobacco use were 23%, 3.2% and 22%, respectively. Overall, the 10-year risk of CVD among patients was <10% in 2778 (97%) patients, from 10% to <20% in 65 (2.3%), from 20% to <30% in 12 (0.4%) and ≥30% in 10 (0.2%).	We have developed a mHealth tool that can be used by CHWs to screen for CVD risk factors, demonstrating the proof of concept in rural Kenya
Vendanthan et al [47]	2019	Community health worker-led program for HTN management	To investigate whether community health workers, equipped with behavioral communication strategies and smartphone technology, can increase linkage of individuals with elevated BP to a hypertension care program in western Kenya and significantly reduce BP.	A total of 1,460 individuals were enrolled (491 usual care, 500 paper-based, 469 smartphone). Average baseline SBP was 159.4 mmHg. Follow-up measures of linkage were available for 1,128 (77%) and BP for 1,106 (76%). Linkage to care was 49% overall, with significantly greater linkage in the usual care and smartphone arms of the trial. Average overall follow-up SBP was 149.9 mmHg. Participants in the smartphone arm had a modestly greater reduction in SBP versus usual care (13.1 mm Hg vs. 9.7 mm Hg).	A strategy combining tailored behavioral communication and mobile health (mHealth) for community health workers led to improved linkage to care, but not statistically significant improvement in SBP reduction.
Vendanthan et al [48]	2020	Nurse-based HTN management program	To evaluate the effect of a nurse-based hypertension management program	The cohort consisted of 1051 adult patients. SBP decreased significantly from baseline to 3 months (nurse-managed patients: slope –4.95 mmHg/month; clinical officer-managed patients: slope –5.28), with no significant difference between groups. DBP also significantly decreased from baseline to 3months with no difference between provider groups.	Nurse-managed hypertension care can significantly improve blood pressure. However, retention in care remains a challenge.
Kengne et al [49]	2009	Nurse-led care for T2DM care	In rural and urban Cameroon, the goal was to develop a protocol-driven primary nurse-led care for type 2 diabetes.	The mean fasting capillary glucose reduced by 1.6 mmol/L (95 percent CI: 0.8-2.3; p or =0.001) between the baseline and final visits. The systolic blood pressure of patients with hypertension reduced dramatically as well. In terms of body weight, no substantial changes were observed.	Nurses may be a viable option for improving diabetes care access in areas where physicians are not available.
Kengne et al [50]	2009	Nurse-led care for hypertension control	To implement a nurse-led protocol for the care of hypertension in Cameroon.	Between baseline and final visits, systolic and diastolic blood pressures dropped by 11.7 mm Hg (95% CI, 8.9-14.4) and 7.8 (95% CI, 5.9-9.6), respectively (P<.001). These changes were consistent in subgroups and after adjustment	Nurse-led clinics can improve hypertension care in both rural and urban areas, but they should be implemented and validated through controlled trials.
Labhardt et al [51]	2010	Non-physician clinicians for treatment of hypertension and diabetes	To examine the effectiveness of integrating care for hypertension and T2DM by task shifting to non-physician clinician (NPC) facilities in eight rural health districts in Cameroon.	Among hypertensive patients with ≥ 2 documented visits (n = 493), systolic BP decreased by 22.8 mmHg (95% CI: -20.6 to -24.9; p < 0.0001) and diastolic BP by 12.4 mmHg (-10.9 to -13.9; p < 0.0001). Among diabetic patients (n = 79) FPG decreased by 3.4 mmol/l (-2.3 to -4.5; p < 0.001).	The integration of hypertension and diabetes into primary health care of NPC facilities in rural Cameroon was feasible in terms of equipment and training, accessible in terms of treatment cost and showed promising BP- and FPG-trends.

Lulebo et al [52]	2017	Nurse-led hypertension management	To investigate the association between task shifting and HTN control in Kinshasa, DRC.	There was no significant difference in the proportion of GRH and HC patients with uncontrolled HTN (76.2% vs 77.7%, p = 0.771). Uncontrolled HTN was associated with co-morbidity (OR = 10.3; 95% CI: 3.8–28.3) and the type of antihypertensive drug used (OR = 4.6; 95% CI: 1.3–16.1).	Uncontrolled HTN was not associated with the type of health facility. This finding suggests that the management of HTN at primary healthcare level might be just as effective as at secondary level.
Hailu et al. [53]	2018	Nurse-led diabetes self-management education	To determine effects of diabetes self-management education on clinical outcomes among T2DM patients in Ethiopia.	Mean HbA1c was significantly reduced by 2.88% within the intervention group and by 2.57% within the comparison group. However, change in the proportion of participants with target HbA1c and end-line mean HbA1c difference between the groups were not significant.	After 9 months of nurse-led DSME, HbA1c was significantly reduced within both groups but there was no significant difference in HbA1c between groups.
Kirwan et al [54]	2016	Nurse-led program to improve early detection of AKI in community health facilities	To assess if a nurse-led program will improve the overall care and understanding of patients with AKI that will still be effective after 3 months.	The nurse-led intervention significantly improved healthcare workers attitudes towards detecting or managing patients with suspected AKI ($p<0.0001$) with significant improvements in the completion of fluid charts and recording of urine output ($p<0.0001$). Knowledge and clinical intervention was still present three months later.	A low cost, nurse-led AKI educational intervention improved the knowledge and management of AKI. The knowledge was still retained at 3-months post training.
Ngoga et al [55]	2019	Nurse-led hypertension treatment program	To describe follow-up and treatment outcomes in stage 1 and 2 hypertension patients receiving care at health centres closer to home in comparison to patients receiving care at DHs further from home	Patients at district hospitals (DH) travelled significantly further to receive care (10.4 km vs. 2.9 km; $P <0.01$). Odds of being retained were significantly lower among DH patients when not adjusting for distance (OR 0.11, $P = 0.01$). The retention effect was consistent but no longer significant when adjusting for distance (OR 0.18, $P = 0.10$). For those retained, there was no significant difference in achieving blood pressure targets between the DHs and HCs.	By removing the distance barrier, decentralizing hypertension management to HCs may improve long-term patient retention and could provide similar hypertension outcomes as DHs.
Stephens et al [56]	2021	Trained village health workers for hypertension and NCD screening.	To assess the role of village health workers (VHW) in screening and managing hypertension and other NCDs at a community level.	Of 4283 people ages 30–69 screened for hypertension, 22% had a blood pressure (BP) ≥140/90 and 5% had a BP ≥ 160/100. All 163 people with SBP ≥170 during door-to-door screening were referred for evaluation, of which 91 (59%) had repeated BP ≥170 and were enrolled in treatment. Of 761 patients enrolled, 413 patients are being treated for hypertension and 68% of these had their most recent blood pressure below the treatment target.	VHWs are able to not only screen patients for hypertension, but also to manage their disease in monthly village-based clinics. Mid-level providers at a local district hospital NCD clinic and faculty from an academic center provide institutional support to VHWs, stream-line referrals for complicated patients and facilitate provider education at all levels of care.

Abbreviations: NCD – non-communicable diseases; SBP – systolic blood pressure; DBP – diastolic blood pressure; AKI – acute kidney injury; T2DM – type 2 diabetes mellitus; HTN – hypertension; CVD – cardiovascular disease; HbA1c – hemoglobin A1C (glycated hemoglobin); FPG – fasting plasma glucose; RR – relative risk.
